# Testing for Ketoprofen Binding to HSA Coated Magnetic Nanoparticles under Normal Conditions and after Oxidative Stress

**DOI:** 10.3390/molecules25081945

**Published:** 2020-04-22

**Authors:** Marta Ziegler-Borowska, Kinga Mylkie, Pawel Nowak, Patryk Rybczynski, Adam Sikora, Dorota Chelminiak-Dudkiewicz, Anna Kaczmarek-Kedziera

**Affiliations:** 1 Faculty of Chemistry, Nicolaus Copernicus University in Torun, Gagarina 7, 87-100 Torun, Poland; kinga.mylkie@o2.pl (K.M.); nowak19981411@wp.pl (P.N.); pat_ryb@doktorant.umk.pl (P.R.); dorotachelminiak@wp.pl (D.C.-D.); teoadk@umk.pl (A.K.-K.); 2 Faculty of Pharmacy, Collegium Medicum in Bydgoszcz, Nicolaus Copernicus University in Torun, dr A. Jurasza 2, 85-089 Bydgoszcz, Poland; sikora.a.e@gmail.com

**Keywords:** human serum albumin (HSA), magnetic nanoparticles, oxidative stress, polysaccharide coating, HSA–drug interaction, ketoprofen

## Abstract

Binding and transport of ligands is one of the most important functions of human blood serum proteins. Human serum albumin is found in plasma at the highest concentration. Because of this, it is important to study protein–drug interactions for this albumin. Since there is no single model describing this interaction, it is necessary to measure it for each active substance. Drug binding should also be studied in conditions that simulate pathological conditions of the body, i.e., after oxidative stress. Due to this, it is expected that the methods for testing these interactions need to be easy and fast. In this study, albumin immobilized on magnetic nanoparticles was successfully applied in the study of protein–drug binding. Ketoprofen was selected as a model drug and interactions were tested under normal conditions and artificially induced oxidative stress. The quality of obtained results for immobilized protein was confirmed with those for free albumin and literature data. It was shown that the type of magnetic core coverage does not affect the quality of the obtained results. In summary, a new, fast, effective, and universal method for testing protein–drug interactions was proposed, which can be performed in most laboratories.

## 1. Introduction

**Figure 1:**
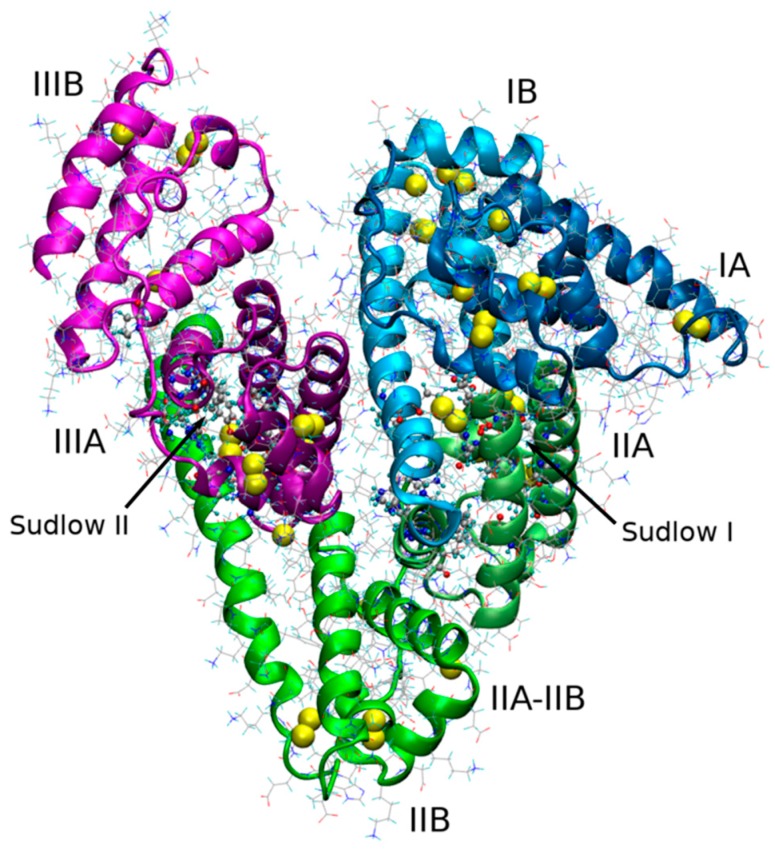
Structure of HSA downloaded from RCSB Protein Data Bank (PDB ID: 1AO6).

Human serum albumin (HSA) is one of the most important globular plasma proteins containing a single heart-shaped polypeptide chain of 585 amino acid residues organized in three homologous domains (I, II and III) with two subdomains, A and B (Figure 1). The most important role of HSA is the ability to bind and transport substances [1]. Compounds bound and transported by albumin are fatty acids, bilirubin, metal ions, and, above all, drugs. In the structure of HSA, two binding sites for drugs can be distinguished: the Sudlow I site located in subdomain IIA and the place of Sudlow II in subdomain IIIA (Figure 1) [2]. Drugs related to HSA are pharmacologically inactive—only the free, unbound fraction of the drug is active and reaching the target causes a pharmacological response of the body and is available to elimination processes [3]. Because the plasma HSA content is high, the study of the interaction of drugs with this protein is very important in pharmacology, especially for newly tested active substances with expected pharmacological activity. There are so many models describing the interaction of HSA with a drug that for each active substance able to bind to albumin this interaction must be qualitatively and quantitatively determined.

As mentioned above, investigation of the interaction of drugs with HSA is crucial not only to understand the structural relationships of protein–ligand complexes but primarily to verify the basic pharmacological parameters and determine the dose of the drug. As the main plasma protein, HSA is sensitive to modifications, in particular in its redox state.

Reactive oxygen species (ROS) are important factors which can change the redox state of albumin. The effect of ROS action on body components such as proteins is called oxidative stress. It accompanies many diseases related to inflammation, autoimmune response, invasive surgery, or even during intensive exercise [4,5,6]. HSA can be modified by the action of oxidative stress at various places in the structure and by different ROS. Due to the kind of oxidant factor, numerous types of free radicals are formed and attack the protein structure. The hydroxyl radical HO^•^ generated upon exposure to ionizing irradiation or reaction of hydrogen peroxide in the presence of Fe^2+^ ions is a strong factor which can attack all amino acid residues in proteins. Another reactive form is the superoxide radical, which leads to the formation of hypochlorous acid (HClO) in vivo. Hypochlorous acid interacts with ammonia and amino groups to form chloramines, which are highly reactive. For the above reason, when investigating the effect of artificially induced oxidative stress, three reactive forms should be taken into account: hydroxyl radical, hydrogen peroxide, and chloramine-T. According to literature reports, aromatic amino acids (tryptophan and tyrosine) and sulfur-containing amino acids (cysteine and methionine) are the places most susceptible to oxidizing agents. Under the conditions of oxidative stress, the formation of dimeric structures of aromatic amino acids and the formation of disulfide bonds between amino acids containing sulfur occurs in these places [7]. Moreover, the polypeptide chain may be denatured. These oxidative modifications of HSA structure may have different consequences for this protein function. As can be expected, structural changes occurring in the HSA molecule under the influence of oxidative stress affect its pharmacokinetic parameters and above all the drugs binding properties.

Because there is no single model describing the drug–HSA interaction under normal conditions, the situation is similar in the case of oxidative stress. The amount of drug bound by oxidized HSA may increase for some active substances and decrease for others; hence, it is necessary to study these interactions for each ligand. In clinical practice, it is known that, in pathological conditions, drug doses due to a change in the amount of HSA-related fraction must be determined individually for a given substance [8]. That is why it is so important that the procedures for testing these interactions are quick and simple.

**Figure 2:**
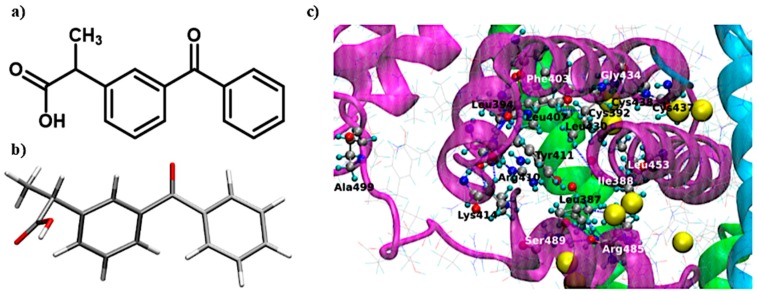
(**a**) Structure of ketoprofen; (**b**) lowest energy conformer of ketoprofen optimized within the B3LYP/6-311++G(d, p) approach in vacuum; and (**c**) Sudlow II site of HSA (PDB ID: 1AO6) with CPK representation for active site residues. The large yellow balls depict sulfur atoms.

Nonsteroidal anti-inflammatory drugs (NSAIDs) are some of the most frequently administered drugs also in diseases with oxidative stress such as rheumatoid arthritis (RA) and postoperative pain. Ketoprofen (KP) is a propionic acid derivative which is available over the counter at lower doses (Figure 2a). This makes it one of the most frequently used NSAIDs. It is known that ketoprofen is bounded by the HSA structure at the Sudlow II site (Figure 2b), which, according to literature reports, is very sensitive to oxidizing agents [1]. The properties of KP and its wide application why in the study of the interaction of the drug–HSA it can be used as a model active substance.

Experimentally, drug interaction with HSA is determined using techniques that allow effective separation of the protein–drug complex from the supernatant, such as ultrafiltration [9], microdialysis, and ultracentrifugation [10]. These methods are relatively difficult and time consuming. For this reason, it is important to develop new methods that would allow the study of these interactions in a simpler and more efficient way. A very good solution may be to apply HSA immobilized on a support allowing for quick and efficient separation of the protein–drug from the supernatant. For this approach, it is necessary to maintain full activity of albumin and its ability to ligand binding. This type of carrier can be magnetic nanoparticles (MNPs) with HSA immobilized on their functional surface.

It is known from our previous studies that the surface of magnetic nanoparticles can be designed and modified at different ways for various application [11,12,13]. In addition, it is proved by an anti-HSA test that HSA immobilized on the surface of MNPs retains its activity [14,15]. Thus, in this article, magnetic nanoparticles coated with modified polysaccharides, which were previously obtained and characterized in our group, were used as a support to determine immobilized HSA–drug interactions [14,15,16]. Application of several materials as carriers that differ in the ability to bind HSA (different number of amine groups on the surface) would allow clarifying whether the type of nanoparticles and the amount of immobilized HSA have an impact on the results of HAS–drug interaction. Ketoprofen (KP) as a model drug from the NSAIDs group was chosen for this investigation. The study of immobilized HSA binding of ketoprofen was carried under normal conditions and after treatment of immobilized HSA with oxidative stress factors: hydroxyl radical, hydrogen peroxide, and chloramine-T. All of the obtained results were compared with native HSA–ketoprofen interaction performed by traditional methods.

Even though there are many reports in the literature on the immobilization of HSA on MNPs, there is only one study in which HSA–MNPs system was used for further research as the study of protein–drug interactions [17]. In the cited work, the interaction of donepezil with HSA physically (not chemically as in our work) immobilized on magnetic nanoparticles was studied.

Thus, the main goal of this work was to develop a new, simple, fast, and effective method in testing the binding of drugs to chemically immobilized human blood serum protein, which allows obtaining repeatable results. These interactions were also investigated under artificially induced oxidative stress conditions. The goal of the current work was to confirm that functionalized magnetic nanoparticles can be a universal carrier for protein in studying these interactions. In addition, the influence of the type of nanoparticles used on the obtained results and their comparability was examined.

## 2. Materials and Methods

### 2.1. Materials

Human Serum Albumin, anti-HSA, ketoprofen, chloramine-T, hydrogen peroxide, iron (II) chloride tetrahydrate, iron (III) chloride hexahydrate, chitosan (low molecular weight), starch from potato, glutaraldehyde, sodium periodate, ethylenediamine, acetic acid, sodium hydroxide, EDC (*N*-(3-dimethylaminopropyl)-*N*′-ethylcarbodiimide hydrochloride), sulfo-NHS (*N*-hydroxy-sulfosuccinimide sodium salt), glycine, isopropanol, acetic acid, ethanol, ninhydrin reagent, and Bradford reagent were purchased from Sigma–Aldrich (Germany) and used without further purification. Solvents, sodium phosphate dibasic dihydrate, orthophosphoric acid solution for phosphate buffer (PBS) preparation, and sodium acetate were purchased from POCh Gliwice (Poland). All solutions were prepared with deionized water.

### 2.2. Methods

#### 2.2.1. UV–Vis Spectroscopy Measurements

The UV–Vis spectra were recorded using UV-1601 PC (Shimadzu, Tokyo, Japan) spectrophotometer in the 200–800 nm range.

##### Calibration Curve for Ketoprofen

Stock solution was prepared by dissolving 12.7 mg ketoprofen in phosphate buffer (PBS, pH 7.4, 50 mM) in a 500 mL volumetric flask. The solution was left at room temperature for 24 h. Then, standard solutions were prepared according to the following procedure. To 10 mL volumetric flasks ketoprofen stock solution was pipetted, respectively, 1.0, 2.0, 3.0, 4.0, 5.0, 6.0, 7.0, 8.0, 9.0, and 10.0 mL of its volume. Then, the flasks were filled to its volume with phosphate buffer and mixed thoroughly. The absorbance was measured at a wavelength of 256 nm against phosphate buffer as a reference sample.

##### Calibration Curve for Human Serum Albumin (HSA)

Five weights of 1–10 mg human serum albumin were prepared. Albumin was suspended in PBS (250 µL) at pH = 7.4 (50 mM). Ten microliters of the resulting suspension were taken to separate Eppendorf tubes and 190 µL of phosphate buffer were added to the protein suspension. Then, 1.5 mL of Bradford reagent were added to each sample. After 15 min, absorbance was measured at 595 nm.

#### 2.2.2. High-Performance Liquid Chromatography (HPLC) Analysis

The Shimadzu HPLC system (Tokyo, Japan) was equipped with two LC-30AD solvent delivery pumps combined with gradient system, a degasser model DGU-20A5, an autosampler model SIL-30AC, a column oven model CTO-20AC and UV detector model SPD-M20A. To achieve satisfactory chromatographic resolution of ketoprofen, the influence of the composing mobile phase, its flow rate, and the type of stationary phase were investigated. Finally, the most appropriate mobile phase consisted of methanol/water/formic acid in ratio 65/35/0.1 (*v/v/v*). The chromatographic process was operated at 30 °C with the use of Kinetex C18 column (250 mm × 4.6 mm × 5 µm). The detection was made with the use DAD detector at 220 nm. The calibration curve was prepared in the range of 2–400 µg/mL and triply repeated. Chromatograms and calibration curve are included in Supplementary Materials (Table S1 and Figure S1).

#### 2.2.3. Computational Methods

The structure of human serum albumin was downloaded from RCSB Protein Data Bank (PDB ID: 1AO6). The hydrogens were added and proper charges were ensured according to pKa of the amino acids in CHARMM force field and corresponding protonation states in pH = 7 were assigned with PROPKA implemented in PDB2PQR Server Version 2.0.0 [18]. The geometry of ketoprofen molecule was fully optimized on its potential energy surface in vacuum within the B3LYP/6-311++G(d,p) approach, as implemented in Gaussian16 [19], and the most stable conformer was chosen for further study. The character of the stationary points was confirmed with the harmonic vibrational analysis. Docking of ketoprofen to the Sudlow II site of HSA was performed with the genetic algorithm implemented within AutoDock 4.2.6 [20]. The number of active torsions of ketoprofen was set to 5 (maximal number available in the rigid drug molecule). HSA was treated as rigid. Absence of solvent molecules was assumed. In total, 100 runs were performed.

### 2.3. Magnetic Nanoparticles Synthesis

#### 2.3.1. Magnetic Nanoparticles Coated with Chitosan and Cross-Linked with Squaric Acid (Fe_3_O_4_–CS(SqA))

Chitosan (0.2 g) was added into 1% acetic acid solution (20 mL) and mechanically stirred at room temperature until solution become homogenous. Iron (II) chloride tetrahydrate (3.7 mmol) and iron (III) chloride hexahydrate (7.5 mmol) were added. Slow addition of droplets of 30% solution of NaOH (15 mL) formed the magnetic nanoparticles. The resulting nanomaterial was filtrated and thoroughly washed with deionized water five times. Then, squaric acid (0.022 g, 0.2 mmol) in ethanol (100 mL) was added and the blend was magnetically stirred at 30 °C for 2 h. The obtained magnetic material was magnetically separated, thoroughly washed with deionized water, and dried by lyophilization.

#### 2.3.2. Magnetic Nanoparticles Coated with Chitosan and Cross-linked with Glutaraldehyde (Fe_3_O_4_–CS(Glu))

Chitosan (0.2 g) was added into 1% acetic acid solution (20 mL) and mechanically stirred at room temperature until solution become homogenous. Iron (II) chloride tetrahydrate (3.7 mmol) and iron (III) chloride hexahydrate (7.5 mmol) were added. Slow addition of droplets of 30% solution of NaOH (15 mL) formed the magnetic nanoparticles. The resulting nanomaterial was filtrated and thoroughly washed with deionized water for five times. Then, 20 mL of 6.5% glutaraldehyde water solution were added, and the mixture was mechanically stirred at room temperature for 30 min. The obtained magnetic material was magnetically separated, thoroughly washed with deionized water, and dried by lyophilization.

#### 2.3.3. Magnetic Nanoparticles Coated with Modified Chitosan with One Long-Distanced Free Amino Group (Fe_3_O_4_ CS–Et(NH_2_))

Chitosan (0.2 g) was added to acetic acid solution (C = 1%, 20 mL) and mechanically stirred at room temperature for 20 min. Iron (II) chloride tetrahydrate (0.74 g, 3.75 mmol) and iron (III) chloride hexahydrate (2.02 g, 7.5 mmol) were added (1:2 molar ratio) and the resulting solution was chemically precipitated at room temperature by adding dropwise 30% solution of NaOH (7 mL). The black mixture was formed, separated by applying magnet, and washed by deionized water five times. Then, 10 mL of bicarbonate buffer pH = 10 and 10 mL of glutaraldehyde solution (5%) were added and the mixture was mechanically stirred at room temperature for 1 h, separated by magnet, and dried under vacuum. Magnetic nanoparticles were pounded with ethylenediamine (2.4 g, 40 mmol) in an agate mortar at room temperature for 1 min without solvent. The resulting magnetic material was washed five times with deionized water and dried by lyophilization.

#### 2.3.4. Magnetic Nanoparticles Coated with Modified Chitosan with Three Long-Distanced Free Amino Group (Fe_3_O_4_–CSEt(NH_2_)_3_)

Chitosan (0.2 g) was added into 1% acetic acid solution (20 mL) and mechanically stirred at room temperature for 20 min. Iron (II) chloride tetrahydrate (0.74 g, 3.75 mmol) and iron (III) chloride hexahydrate (2.02 g, 7.5 mmol) were added (1:2 molar ratio) and the resulting solution was chemically precipitated at room temperature by adding dropwise 30% solution of NaOH (7 mL). The black mixture was formed and epichlorohydrin (0.2 mL, 2.5 mmol) was added and the mixture was stirred at 50 °C for 2 h. After cooling to room temperature, sodium periodate solution (0.16 g in 2.5 mL of water) was added and the mixture was stirred for 30 min. The black precipitate was separated by filtration and washed by deionized water for five times. Then, 10 mL of bicarbonate buffer pH = 10 and 10 mL 5% glutaraldehyde solution were added, and the mixture was mechanically stirred at room temperature for 1 h, separated by magnet, and dried under vacuum. Magnetic nanoparticles were pounded with ethylenediamine (2.4 g, 40 mmol) in an agate mortar at room temperature for 1 min without solvent. The resulting magnetic material was washed five times with deionized water and dried by lyophilization.

#### 2.3.5. Magnetic Nanoparticles Coated with Aminated Starch (Fe_3_O_4_–AS)

Starch (2 g) was dissolved in water (40 °C). Then, iron (II) chloride tetrahydrate (3.7 mmol) and iron (III) chloride hexahydrate (7.5 mmol) were added and the resulting solution was precipitated by adding dropwise 30% solution of NaOH (15 mL). The black mixture was recovered from the suspension by applying a magnet, washed with deionized water, and then dispersed in water under mechanical stirring. Sodium periodate solution (0.7 M, 5 mL) was added to the MNPs suspension under magnetic stirring. The mixture was heated to 40 °C and stirring was continued at this temperature for 1 h. After cooling to room temperature MNPs were isolated by applying a magnet, washed three times with deionized water and dried under vacuum (30 °C, 24 h). Obtained MNPs (0.5 g) were pounded with ethylenediamine (0.02 mL) in an agate mortar at room temperature for 1 min without solvent. The black solid was washed three times with deionized water and dried by lyophilization.

### 2.4. Quantification of Available Primary Amino Groups on Magnetic Nanoparticles Surface

The amount of primary amino groups on the magnetic nanoparticles were estimated by the ninhydrin method. The calibration curve was prepared using glycine as a standard ranging from 0.6 to 2 mM in 0.1 mM acetate buffer of pH 5.5. The ninhydrin reagent (2 mL) was added to 2 mL solution of each concentration of glycine and well mixed. The blank reagent was composed with 2 mL of distilled water and 2 mL of ninhydrin reagent. The suspensions of magnetic nanoparticles were obtained by dispersing of 10 mg of synthesized nanoparticles in 2 mL of 0.1 mM buffer acetate (pH 5.5) and then 2 mL of ninhydrin reagent were added. All standard curve solutions and suspensions of nanoparticles were capped, mixed by hand, and heated in boiling water for 15 min. After cooling, 3 mL of 50% ethanol were added to each tube. The concentration of primary amino groups was determined with the use of spectrophotometric measurements at 570 nm.

### 2.5. Human Serum Albumin Immobilization

To 10 mg of HSA in 1 mL of 50 mM PBS (pH 6.5), EDC (2 mg) was added and incubated at 21 °C for 1 h. Then, sulfo-NHS (2.4 mg) in 50 μL of 50mM PBS was added and incubated as previously (21 °C, 1 h). After incubation, magnetic nanoparticles (50 mg) were added and incubated for 2 h at 21 °C. Next, 2 mg of ethanolamine were added to saturate unbound EDC/NHS and the mixture was incubated at 21 °C for next 5 min. Then, the supernatant was removed by applying a magnet and the amount of immobilized HSA bounded on magnetic nanoparticles surface was determined by the Bradford protein assay method. A calibration curve (R^2^ = 0.998) constructed with analyzed protein solution of known concentration (0.1–1.1 mg/mL) was used in the calculation of albumin concentration. All data used for calculation are the average of triplicate experiments. The activity agglutination test was performed for HSA-coated magnetic nanoparticles on the test glass slide for 10 μL of nanoparticles and 10 μL of HSA solution in PBS (pH = 7.4). The aggregation ability was estimated. A control test for nanoparticles without HSA was also made.

### 2.6. The Interaction of HSA with Ketoprofen without Oxidative Stress

#### 2.6.1. The Interaction of Native HSA with Ketoprofen

A solution of human serum albumin was prepared in 50 mM PBS at pH 7.4 with a concentration of C_HSA_ = 2.5 μM. To 1 mL of this HSA solution, 1 mL of 22.88 mg/L ketoprofen solution (PBS, pH 7.4) solution was added and incubated in Thermomixer at 20 °C for 2 h. After incubation, the mixture from each Eppendorf tubes was centrifuged at 15,000× *g* for 30 min at 20 °C. The supernatant was separated by microfiltration, and its absorbance was measured at 256 nm. A calibration curve (R^2^ = 0.9997) constructed with analyzed ketoprofen solution of known concentration (2.54–25.43 mg/L) was used in the calculation of binding amount ketoprofen with protein. All data used for calculation are the average of triplicate experiments.

#### 2.6.2. The interaction of HSA immobilized on Magnetic Nanoparticle (MNP) Surface with Ketoprofen

To 10 mg of magnetic nanoparticles with immobilized human albumin 2 mL of 14.56 mg/L ketoprofen solution (PBS, pH 7.4) was added and incubated in Thermomixer at 20 °C for 2 h. Then, the supernatant was separated by applying a magnet and its absorbance was measured at 256 nm. A calibration curve (R^2^ = 0.9997) constructed with analyzed ketoprofen solution of known concentration (2.54–25.43 mg/L) was used in the calculation of binding amount ketoprofen with protein immobilized on MNPs. All data used for calculation are the average of triplicate experiments.

### 2.7. The Interaction of HSA with Ketoprofen under Oxidative Stress Conditions

#### 2.7.1. The Interaction of Oxidative HSA with Ketoprofen—H_2_O_2_

To 1 mL human serum albumin solution (C_HSA_ = μM 2.5, 1.663 mg HSA, PBS, pH 7.4), 200 mM hydrogen peroxide (1 mL) was added and incubated in Thermomixer at 20 °C for 2 h. After incubation, the mixture from each of Eppendorf tubes was centrifuged at 15,000× *g* for 30 min at 20 °C. The supernatant was removed and 1.5 mL of 14.56 mg/L ketoprofen solution (PBS, pH 7.4) was added to the protein suspension. The mixture was incubated at 20 °C for 2 h in Thermomixer. After incubation, the supernatant was separated by microfiltration, and its absorbance was measured at 256 nm. All data used for calculation are the average of triplicate experiments.

#### 2.7.2. The Interaction of Oxidative HSA with Ketoprofen—Hydroxyl Radical

To 1 mL human serum albumin solution (C_HSA_ = μM 2.5, 1.663 mg HSA, PBS, pH 7.4), 200 mM hydrogen peroxide (1 mL) and 50 μL of 10 mM iron (II) chloride solution were added and incubated at 20 °C for 2 h in Thermomixer. After incubation, the mixture from each of the Eppendorf tubes was centrifuged at 15,000× *g* for 30 min at 20 °C. The supernatant was removed and 1.5 mL of 14.56 mg/L ketoprofen solution (PBS, pH 7.4) were added to the protein suspension. The mixture was incubated at 20 °C for 2 h in Thermomixer. After incubation, the supernatant was separated by microfiltration, and its absorbance was measured at 256 nm. All data used for calculation are the average of triplicate experiments.

#### 2.7.3. The Interaction of Oxidative HSA with Ketoprofen—Chloramine-T

To 1 mL human serum albumin solution (C_HSA_ = μM 2.5, 1.663 mg HSA, PBS, pH 7.4), 10 mM chloramine-T (1 mL) was added and incubated at 20 °C for 2 h. After incubation, the mixture from each of the Eppendorf tubes was centrifuged at 15,000× *g* for 30 min at 20 °C. The supernatant was removed and 1.5 mL of 14.56 mg/L ketoprofen solution (PBS, pH 7.4) was added to the protein suspension. The mixture was incubated at 20 °C for 2 h in Thermomixer. After incubation, the supernatant was separated by microfiltration, and its absorbance was measured at 256 nm. All data used for calculation are the average of triplicate experiments.

#### 2.7.4. The Interaction of Oxidative HSA Immobilized on MNPs Surface with Ketoprofen—H_2_O_2_

To 10 mg of magnetic nanoparticles with immobilized HSA, 0.6 mL of 200 mM hydrogen peroxide were added for each milligram of immobilized HSA (Table 1) and incubated at 20 °C for 2 h in Thermomixer. Then, the supernatant was removed by applying a magnet and 2 mL of 14.56 mg/L ketoprofen solution (PBS, pH 7.4) were added to the suspension. The mixture was incubated in Thermomixer at 20 °C for 2 h. After incubation, the supernatant was separated by applying a magnet and its absorbance was measured at 256 nm. All data used for calculation are the average of triplicate experiments.

#### 2.7.5. The Interaction of Oxidative HSA Immobilized on MNPs Surface with Ketoprofen—Hydroxyl Radical

To 10 mg of magnetic nanoparticles with immobilized HSA, 0.6 mL of 200 mM hydrogen peroxide and 30 μL of 10 mM iron (II) chloride solution were added for each milligram of immobilized HSA (Table 1) and incubated at 20 °C for 2 h in Thermomixer. Then, the supernatant was removed by applying a magnet and 2 mL of 14.56 mg/L ketoprofen solution (PBS, pH 7.4) were added to the suspension. The mixture was incubated in Thermomixer at 20 °C for 2 h. After incubation, the supernatant was separated by applying a magnet and its absorbance was measured at 256 nm. All data used for calculation are the average of triplicate experiments.

#### 2.7.6. The Interaction of Oxidative HSA Immobilized on MNPs Surface with Ketoprofen—Chloramine-T

To 10 mg of magnetic nanoparticles with immobilized HSA, 10 mM chloramine-T (0.6 mL) was added for each milligram of immobilized HSA (Table 1) and incubated at 20 °C for 2 h in Thermomixer. Then, the supernatant was removed by applying a magnet and 2 mL of 14.56 mg/L ketoprofen solution (PBS, pH 7.4) was added to the suspension. The mixture was incubated in Thermomixer at 20 °C for 2 h. After incubation, the supernatant was separated by applying a magnet and its absorbance was measured at 256 nm. All data used for calculation are the average of triplicate experiments.

### 2.8. Isotherms of a Binding of KP to HSA (Fe_3_O_4_–AS–HSA) and Oxidized HSA with Chloramine-T (Fe_3_O_4_–AS–oHSA, Chloramine-T)

One milliliter of ketoprofen solution with various concentrations (0.02288, 0.02034, 0.01780, 0.01271, 0.01017, 0.00763, 0.00509, and 0.00255 mg/mL (PBS, pH 7.4)) was added to separate Eppendorf tubes containing 10 mg of magnetic nanoparticles: Fe_3_O_4_–AS–HSA or Fe_3_O_4_–AS–oHSA, Chloramine-T. The mixture was incubated in Thermomixer at 20 °C for 2 h. After incubation, the supernatant was separated by applying a magnet and its absorbance was measured at 256 nm. All data used for calculation are the average of triplicate experiments.

## 3. Results and Discussion

### 3.1. Immobilization of HSA on Magnetic Nanoparticles Surface

**Figure 3:**
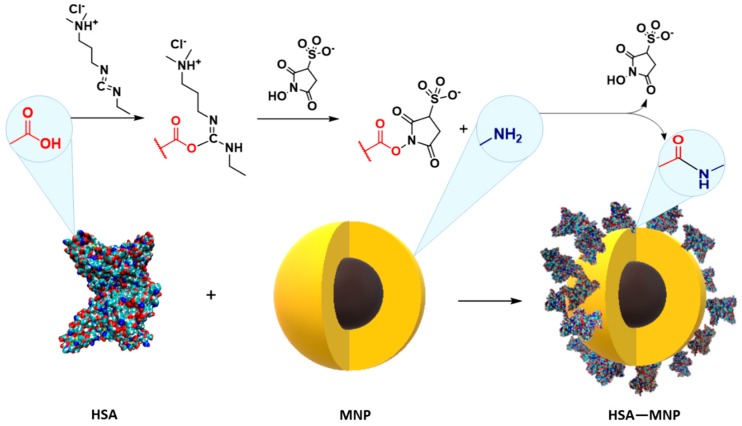
Scheme of human serum albumin (HSA) immobilization on magnetic nanoparticles (MNPs) surface.

One of the main factors determining the amount and activity of proteins immobilized on the carrier is the number of functional groups on the support surface able to react with protein amino or carboxyl moieties [14,21]. In this work magnetic nanoparticles (MNPs) were used as the support. The most commonly applied method of protein chemical immobilization on the MNPs is the use of its carboxyl groups with a mixture of EDC and sulfo-NHS as a binding agent and reaction with external amino groups of the carrier surface (Figure 3). Based on our experience, for the EDC/sulfoNHS immobilization of HSA, magnetic nanoparticles coated with polysaccharides with different content of reactive amino groups were chosen.

**Figure 4:**
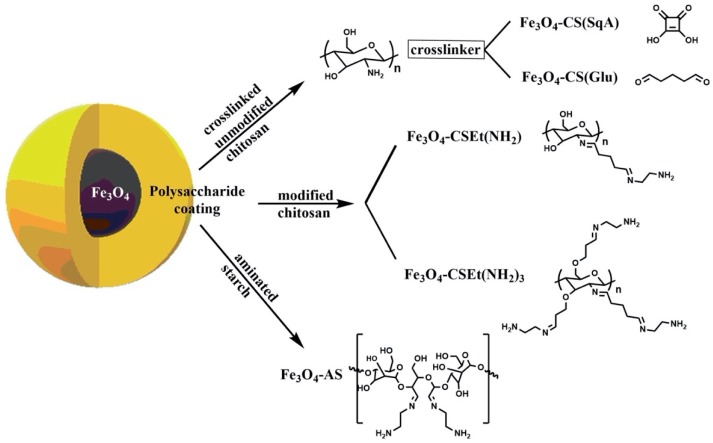
Scheme magnetic nanoparticles coated with different polysaccharide type used for HSA immobilization.

Due to the influence of the amount of free amino groups on the surface of the support on the protein immobilization efficiency and drug binding ability it was decided to use several variants of nanoparticles (Figure 4). First, nanoparticles coated with unmodified chitosan containing one free amino group in a saccharide unit were selected (Figure 4). Moreover, two types of polymer chain crosslinker were also used for this chitosan coating: glutaraldehyde (Fe_3_O_4_–CS(Glu)) and the squaric acid (Fe_3_O_4_–CS(SqA)) This allowed investigating whether the crosslinking agent has an impact on immobilization efficiency and HSA binding activity. Both types of nanoparticles are characterized by medium content of free amine groups on the surface (Table 2).

Another group of applied nanoparticles were MNPs coated with chemically modified chitosan where the external amino groups were distanced from the polysaccharide matrix (Figure 4). This approach was used to check whether the distance of immobilized HSA on MNPs would affect its interaction with ketoprofen. Nanoparticles with one Fe_3_O_4_–CSEt(NH_2_) and three Fe_3_O_4_–CSEt(NH_2_)_3_ distanced amino groups with the content of these groups on the surface 3.15 and 8.34 mM/g, respectively, were selected (Table 2).

One can notice that modified chitosan with two distanced amino groups was not used for this research, although it was previously synthesized by our group [14]. Unfortunately, according to our earlier studies, these nanoparticles are not suitable for effective immobilization of HSA. To replace this material with carriers that allow effective protein binding as well as preserve the increasing number of amino groups on support surface, nanoparticles coated with aminated starch (Fe_3_O_4_–AS) with 5.63 mM/g external amino groups content were selected (Figure 4 and Table 2) [15]. All used nanoparticles were dried by lyophilization, which has been shown in previous studies to be important for the effectiveness of HSA immobilization [14].

As expected and partly previously reported, the amount of bounded albumin increases with the content of free amino groups on the surface of the magnetic support and distance from the polymer matrix in contrast to the type of crosslinker (Table 2) [14,15]. For all nanomaterials with bounded HSA, an anti-HSA aggregation test was performed to confirm albumin activity [15,22].

The use of these five materials as HSA immobilization carriers determined whether the type of carrier, the amount of immobilized protein, and its distance from the surface have an impact on the results of the HSA–drug interaction. Considering the impact of these factors would allow the development of a quick and easy universal method for immobilized protein–drug interaction measurement, which, as mentioned above, was the main goal of this study.

### 3.2. Interaction of HSA with KP without Oxidative Stress

**Figure 5:**
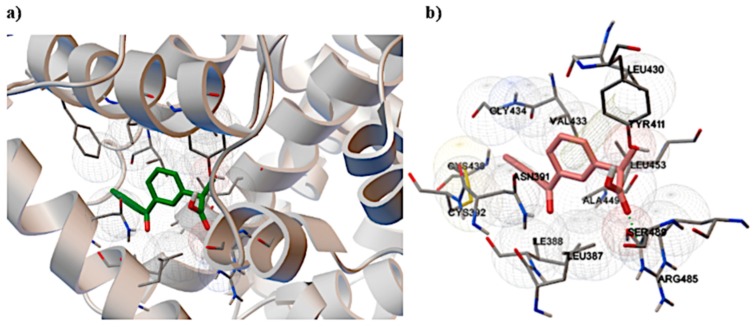
Ketoprofen docked into the Sudlow II site in HSA by AutoDock.

Ketoprofen, similar to other active substances of an acidic nature, binds to HSA at the Sudlow II site (Figure 5). After binding of KP by HSA, a change in polarity in the HSA peptide chains and, as a result, small but noticeable changes in the secondary structure of the protein were observed. Furthermore, according to the literature, HSA binding of acidic drugs such as KP causes an increase in the amount of α-helix in the albumin structure [23]. These changes in the protein structure after different drug concentration binding can be observed in the form of changes in the emission spectrum of the protein solution; therefore, spectrofluorimetry is the most commonly used method to determine the amount of drug bound to a protein. This method is good for a standard approach, as the interaction of free albumin with a drug and changes in the protein emission spectrum after drug binding are clear and visible. Then, after centrifugation or ultrafiltration, it is possible to measure the emission spectrum of the protein fraction with the adsorbed drug.

The second approach may be to measure the amount of unbounded drug fraction to describe protein–drug interactions. This method is more flexible and versatile because it allows measuring the interactions of a protein with, e.g., a mixture of drugs and applying a simple and available UV–Vis spectroscopy [24]. Unfortunately, the serious limitation for this approach is the difficulty in separating the HSA–drug complex from the supernatant containing the free fraction of active substance. Methods such as centrifugation and ultrafiltration are time consuming and do not guarantee that there is no protein left in the supernatant. Therefore, this approach is rarely used. A very good solution would be a method offering easy and efficient separation of the HSA–drug complex from the supernatant, such as the HSA-coated magnetic material proposed in this paper.

#### 3.2.1. Interaction of Free HSA with KP

It is important that the results obtained for the immobilized HSA–drug interactions test are comparable to those obtained for free HSA. Therefore, the HSA–KP interaction was firstly measured for free, non-immobilized HSA as a reference. As mentioned above, it was decided to focus on measuring the unbound amount of drug in the supernatant to determine the degree of bounded KP using UV–Vis spectroscopy. This approach was also used for free albumin.

The amount of ketoprofen bounded to HSA was determined based on the difference between the amount of KP introduced and the amount of free KP in the supernatant after through centrifugation of the HSA–KP complex. The centrifugation accuracy was confirmed by the lack of absorption in the HSA-specific region in the supernatant. The results were calculated for 1 g of protein with Equation (1):

*b* = (*m*_0_ − *m_s_*)/*m_HSA_* (1)

where *m*_0_ is initial ketoprofen mass, *m_s_* is the mass of ketoprofen in the supernatant after protein interaction, and *m_HSA_* is the mass of used human serum albumin.

**Figure 6:**
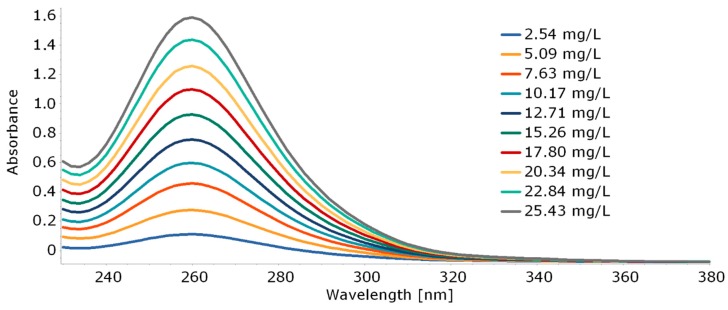
Absorption spectra of ketoprofen with increasing concentrations.

For this purpose, a calibration curve was prepared for KP based on the intensity with a maximum absorbance of 260 nm (Figure 6). The *m_s_* value was read directly from the prepared curve based on this maximum. To confirm the effectiveness of the proposed method, the amount of ketoprofen in the supernatant (*m_s_*) was also determined by HPLC where a calibration curve was prepared for KP concentrations in the range of 1.5–400 mg/L (Table S1 and Figure S1). The results obtained for the interaction of free HSA with ketoprofen are shown in Table 3.

As can be seen, the results obtained on the basis of absorbance measurement by UV–Vis spectrometry and after HPLC analysis are similar and comparable to the literature [25,26]. Therefore, determination of the amount of bound/unbound drug by UV–Vis spectroscopy is reliable and reasonable for further research.

#### 3.2.2. Interaction of Immobilized HSA with KP

**Figure 7:**
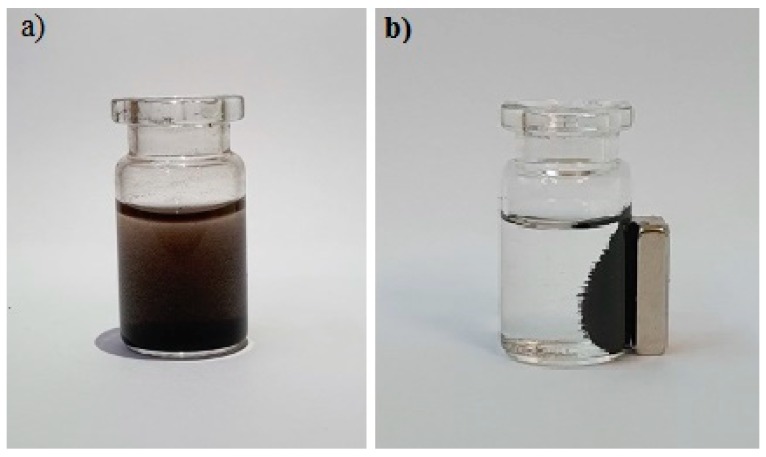
Magnetic nanoparticles after binding ketoprofen with HSA: (**a**) before applying the magnet; and (**b**) after applying the magnet.

The next step was determination of the interaction of ketoprofen with HSA immobilized on magnetic nanoparticles. As for free, non-immobilized HSA, the protein binding effect of ketoprofen was evaluated by measuring the UV–Vis spectrum of the supernatant containing unbound drug. The difference was to skip centrifugation to separate the HSA–KP complex, which was replaced by separating the HSA–KP coated nanoparticles from the supernatant by using a magnet (Figure 7).

This allowed for a significant simplification of the procedure and reduced analysis time. The amount of unbound ketoprofen was determined in the supernatant using UV–Vis spectroscopy and the amount of protein-bound ketoprofen was calculated using Equation (1). Because the results obtained for the amount of unbound ketoprofen with free HSA by measuring absorbance and by HPLC were in agreement, for immobilized HSA, the results were based only on measurements of the absorption spectrum. As in the case of free HSA, the results are given in milligrams of bounded ketoprofen per gram of protein (*b*) (Table 4).

Since the used nanoparticles contain different amounts of immobilized HSA, milligrams of bounded ketoprofen per gram of protein (*b*) is the only parameter that can be compared (Table S2). To determine the degree of drug binding or percentage, the results for individual nanoparticles should be considered. As discussed above, the main goal was to develop a repeatable and universal method for determining drug–protein interaction; thus, a procedure that allows obtaining similar results for the used nanoparticles per gram of HSA was sought. The results of the amount of ketoprofen bound to HSA immobilized on nanoparticles per gram of protein are consistent with the result obtained for native HSA and comparable for each of the used nanomaterials (Table 4).

Thus, in the case of immobilization of HSA on magnetic nanoparticles, the type of support does not affect the result of testing the interaction of the immobilized protein with the drug. Therefore, magnetic nanoparticles can be successfully used as a carrier to determine the ability of the active substance to interact with the protein, regardless of the type of coating of the magnetic core.

### 3.3. Interaction of HSA with KP under Oxidative Stress Conditions

The next step was the use of HSA-coated magnetic nanoparticles to study the interaction of ketoprofen as a model active substance with HSA after oxidative stress. As mentioned above, oxidative stress accompanies many diseases and disrupts the distribution and availability of the drug due to changes in the drug–protein interaction compared to that in stress-free conditions. Most reported studies on the drug binding by oxidized HSA were performed using in vitro oxidation with different methods. Hydrogen peroxide, hydroxyl radical, and chloramine-T were used as well as stress factors. As discussed in the Introduction, it is a simulation of the conditions of in vivo oxidative stress. For comparison, studies were also performed with non-immobilized free albumin as in the case of interaction without a stress factor.

**Figure 8:**
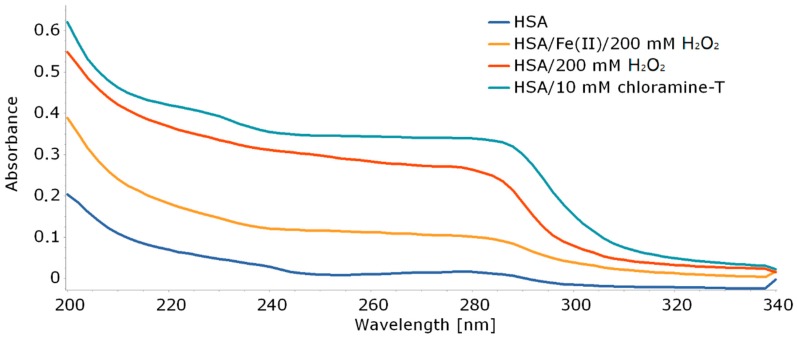
Absorption spectra of nonmodified HSA and HSA stressed with different stress factor.

Because most changes in the structure of HSA under the influence of oxidative stress occur on tryptophan (Trp), tyrosine (Tyr), and cysteine (Cys), it is possible to observe them using UV–Vis spectroscopy (Figure 8) [27].

There are visible changes in the absorption spectrum for HSA oxidized with various stress factors. The most common changes occur in the oxidation of HSA with Chloramine-T. The spectrum of HSA treated with this factor shows a clear increase in absorbance between 310 and 240 nm. According to the literature, these are due to differences in protein fragments containing aromatic amino acids [28]. There are also noticeable changes in the 200–240 nm range due to changes in amino acid residues containing sulfur atoms [27,28]. Aromatic tryptophan as well as cysteine containing sulfur atoms are found in the ketoprofen binding Sudlow II site (Figure 5). Therefore, it can be expected that KP will have difficulty accessing the binding site after oxidation of albumin, which would increase its free concentration in the supernatant. In addition, based on the obtained spectrum, it can be expected that changes in the amount of bound ketoprofen will be most apparent for HSA oxidized with chloramine-T.

Many theories have been used to describe changes under the influence of oxidative stress and their effect on the binding of drugs by proteins; however, there is really no universal model to predict how oxidative stress will affect these parameters for any active substance. Therefore, it is important to study the effect of stress on drug–protein binding for each of active substances [29]. 

For oxidizing albumin immobilized on nanoparticles, HSA-coated MNPs were treated with different stress factors similar to the free HSA. After incubation with oxidant, HSA-coated magnetic nanoparticles were easily separated by applying a magnet. To check whether the conditions of oxidative stress caused the polymer coating with immobilized HSA layer to detach, an absorption spectrum was obtained for the supernatant in which no protein band was found. This confirms that MNPs are stable under conditions of induced oxidative stress. For separated nanoparticles with immobilized oxidized HSA, interaction with ketoprofen was determined exactly in the same way as for the material without oxidative stress.

Table 5 shows the results of a ketoprofen binding study on oxidation-modified HSA immobilized on MNPs and for comparison with free oxidized protein with these same conditions. Complete interaction results with the m*_0_* and m*_s_* value are included in Tables S3–S5.

As can be seen, oxidative stress increases the free ketoprofen fraction for each of the stress factor. These results are in line with literature results obtained with other methods such as spectrofluorimetric measurements, HPLC, circular dichroism, electrophoresis, and nuclear magnetic resonance [29]. In all these works, an increase in the free ketoprofen fraction was observed after interaction with oxidized HSA compared to the unmodified protein.

The amounts of drug-bound protein per gram of HSA (*b*) are comparable with the results for free HSA for all used nanocarriers. As for interaction under stress-free conditions, the polysaccharide coating surrounding the magnetite core does not affect the results obtained for the immobilized HSA–drug system. Therefore, it can be said that any nanoparticles having amino groups capable of immobilizing HSA by EDC/NHS can be used as a carrier in protein–drug interaction studies in conditions with and without oxidative stress.

Changes in the amount of immobilized protein-associated ketoprofen for such stress factors as hydrogen peroxide or hydroxyl radical are comparable. This is due to the similar activity of both factors. Compared with unoxidized HSA, modified protein binds less ketoprofen. The difference is about 0.5 mg for the hydroxyl radical and 1 mg per gram of HSA when using hydrogen peroxide as a stress factor. It is different when albumin is modified with chloramine-T. Here, the change in the amount of bound ketoprofen is about 3.5 mg/g HSA compared to unmodified protein. This means that albumin after stress caused by this factor binds about 50% less drug, and therefore the free and active ketoprofen fraction is half as high as under stress-free conditions. The obtained results are similar to those described in the literature [29]. Such a strong effect of chloramine-T on the binding properties of HSA is explained by the strong reactivity of this factor with the cysteine (Cys) and methionine (Met) sulfhydryl groups, which leads to the formation of disulfide bridges hindering drug access to the binding site as it was already mentioned [30]. 

**Figure 9:**
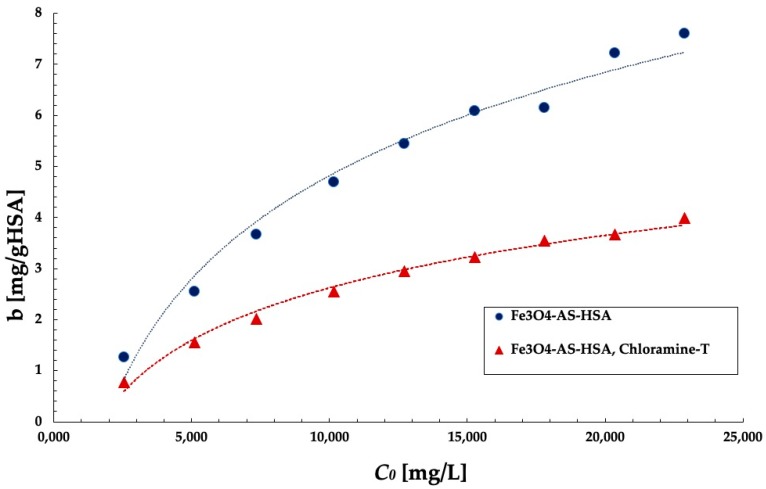
Binding isotherms of Fe_3_O_4_–AS–HSA before and after oxidative stress induced with chloramine-T. Error bars are smaller than the symbols.

Isotherm of binding ketoprofen to Fe_3_O_4_–AS–HSA was obtained as function f(*C_0_*) = *b*, where *C_0_* is initial ketoprofen concentration (mg/L) and *b* is the amount of ketoprofen (mg) bounded to 1 g HSA (Figure 9 and Table 6). Figure 9 shows the curve for immobilized HSA after oxidative stress induced only by chloramine-T due to the largest changes in ketoprofen binding capacity. Both curves for Fe_3_O_4_–AS–HSA before and after chloramine-T induced oxidative stress have a similar shape.

As can be deduced from the analysis of the binding isotherm, the amount of ketoprofen bound to HSA per 1 g of protein increases with increasing of the initial ketoprofen concentration. At the same time, the percentage of drug bound by HSA decreases as the initial concentration of ketoprofen increases—this is associated with less drug availability to the protein binding site (Table 6).

During the oxidative modification of immobilized albumin, it was monitored whether oxidative stress conditions caused damage/detachment of the polysaccharide coating with bound HSA. It was monitored whether a protein absorption band appeared in the supernatant. Its absence clearly indicates that the applied conditions of oxidative stress did not destroy the polysaccharide coating with immobilized HSA on the surface of the nanoparticle.

## 4. Conclusions

In this study, magnetic nanoparticles with human serum albumin immobilized on the surface were for the first time successfully applied for the interaction of HSA–drug determination. As a model drug widely used in different diseases, ketoprofen was chosen. Interactions were measured without HSA oxidation and in artificially induced oxidative stress, which simulate pathological conditions in the body. The use of HSA immobilized on magnetic nanoparticles has significantly facilitated the method of studying protein–ligand interactions due to the easy, fast, and efficient separation of the HSA–drug complex from the supernatant. As a result, it also allowed the use of UV–Vis spectroscopy for quantitative measurement of these interactions, which is a cheaper and more accessible method than commonly used spectrofluorimetry, HPLC, or capillary electrophoresis. The results obtained for the HSA–ketoprofen interactions for the immobilized protein are very similar to those obtained for the free protein both without and after oxidative stress. This shows that HSA immobilized on magnetic nanoparticles can be successfully used to quantify protein–drug interactions. The amounts of ketoprofen remaining after interaction with HSA in free, unbound form are close to the values described in the literature for these interactions.

Oxidative stress was induced by oxidizing agents commonly used in the literature, such as hydrogen peroxide, hydroxyl radical, and chloramine-T. As expected based on changes in HSA under the influence of these factors observed in the absorption spectrum of the protein, the largest differences in the amount of bound ketoprofen after oxidative stress are in the case of oxidation with chloramine-T. The amount of ketoprofen bound by HSA with oxidized chloramine-T is two-fold lower than for unmodified protein. The results are consistent for HSA free and immobilized on nanoparticles and similar with literature reports, thus the approach used in this study is reliable and can be successfully used for such applications.

Moreover, it was also shown that the type of applied nanoparticles does not affect the amount of ketoprofen bound by HSA, which means that, for the immobilization of HSA for quantitative measurement of drug binding, any magnetic carrier having on the surface the group able to immobilize proteins can be used. The polysaccharide shell surrounding the magnetic core of nanoparticles to which HSA is directly bound is stable under conditions of oxidative stress. This means that oxidizing agents only cause HSA oxidative changes without damaging the nanoparticle coating or protein desorption.

As mentioned in the Introduction, the determination of drug binding to human blood serum proteins occurs in different ways and at different degrees for each drug. Therefore, it is necessary in particular for newly synthesized active substances to study this interaction. That is why it is so important that the procedure is simple, fast, effective, and able to be performed in most laboratories. The method proposed in this publication gives such possibilities. The procedure is uncomplicated and UV–Vis spectrometry—to which most laboratories have access—is used for measurements. In addition, the proposed method allows the study of interactions with HSA for a mixture of active substances since the basis for calculating the amount of drug bound to the protein is to measure the amount of its free fraction in the supernatant. The subject for further research is to develop a method for kinetic studies of these interactions with HSA immobilized on MNPs, which is being developed by our team.

## Figures and Tables

**Table 1:** Volume of stress factor solutions used per 10 mg of nanoparticles. Solution concentrations: Chloramine T C = 10 mM, Hydrogen peroxide C = 200 mM, and Iron (II) chloride C = 10 mM.

Nanoparticles (10 mg)	Stress Factor Solution Volume			
	Hydrogen Peroxide (mL)	Hydroxyl Radical		Chloramine-T (mL)
		H_2_O_2_ (mL)	FeCl_2_ (μL)	
Fe_3_O_4_–CS(Glu)–HSA	0.45	0.45	22.0	0.45
Fe_3_O_4_–CS(SqA)–HSA	0.48	0.48	24.0	0.48
Fe_3_O_4_–CSEt(NH_2_)–HSA	0.90	0.90	45.0	0.90
Fe_3_O_4_–AS–HSA	1.00	1.00	50.0	1.00
Fe_3_O_4_–CSEt(NH_2_)_3_–HSA	1.26	1.26	63.0	1.26
HSA	1.00	1.00	50.0	1.00

**Table 2:** Amount of primary amino groups on the MNPs surface and HSA loading (mg/g).

Nanoparticles Type	Amount of NH_2_ Groups (mM/g)	HSA Loading (mg/g)
Fe_3_O_4_–CS(Glu)	3.73	75.56
Fe_3_O_4_–CS(SqA)	3.31	79.16
Fe_3_O_4_–CSEt(NH_2_)	3.15	150.02
Fe_3_O_4_–AS	5.63	165.32
Fe_3_O_4_–CSEt(NH_2_)_3_	8.34	210.00

**Table 3:** Results of free HSA interaction with KP solution detected by UV–Vis spectroscopy and HPLC analysis.

Method of *m_s_* Determination	HSA (g)	*m_0_* (mg)	*m_s_* (mg)	*b* (mg/g HSA)
UV–Vis	0.00250	0.02288	0.00388	7.60
HPLC	0.00250	0.02288	0.00383	7.62

**Table 4:** Results of immobilized HSA interaction with KP solution under normal conditions.

Nanoparticles	HSA (g)	*m_0_* (mg)	*m_s_* (mg)	*b* (mg/g HSA)
Fe_3_O_4_–CS(Glu)–HSA	0.00076	0.02288	0.01713	7.60
Fe_3_O_4_–CS(SqA)–HSA	0.00079	0.02288	0.01687	7.60
Fe_3_O_4_–CSEt(NH_2_)–HSA	0.00150	0.02288	0.01148	7.60
Fe_3_O_4_–AS–HSA	0.00165	0.02288	0.01032	7.60
Fe_3_O_4_–CSEt(NH_2_)_3_–HSA	0.00210	0.02288	0.00692	7.60

**Table 5:** Results of immobilized HSA interaction with KP solution after oxidative stress induced with different stress factors.

Nanoparticles	Stress Factor		
	Hydrogen Peroxide	Hydroxyl Radical	Chloramine-T
	*b* (mg/g HSA)	*b* (mg/g HSA)	*b* (mg/g HSA)
**Fe_3_O_4_–CS(Glu)–HSA**	6.12	6.98	4.07
**Fe_3_O_4_–CS(SqA)–HSA**	6.08	6.56	3.95
**Fe_3_O_4_–CSEt(NH_2_)–HSA**	6.10	6.72	4.03
**Fe_3_O_4_–AS–HSA**	6.08	6.80	3.99
**Fe_3_O_4_–CSEt(NH_2_)_3_–HSA**	6.11	6.78	4.01
**HSA**	6.04	6.75	4.00

**Table 6:** Amount of bounded KP: (mg/gHSA and percent) for different initial KP concentration (mg/L).

KP Initial Concentration (mg/L)	Nanoparticles			
	Fe_3_O_4_–AS–HSA		Fe_3_O_4_–AS–oHSA, Chloramine-T	
	*b* (mg/g HSA)	Bounded KP (%)	*b* (mg/g HSA)	Bounded KP [%]
22.88	7.60	54.91	3.99	28.77
20.34	7.22	58.57	3.67	29.77
17.80	6.15	57.01	3.55	32.91
15.26	6.08	65.74	3.23	34.92
12.71	5.45	70.75	2.95	38.30
10.17	4.70	76.25	2.55	41.37
7.63	3.67	79.36	2.01	43.47
5.09	2.55	82.50	1.55	50.15
2.55	1.27	82.18	0.77	49.82

## Supplementary Materials

The following are available online: Figure S1: Ketoprofen chromatograms, Table S1: Results of HPLC analysis for ketoprofen calibration curve, Table S2: Results of ketoprofen interaction with HSA without oxidative stress, Table S3: Results of HSA–ketoprofen interactions under oxidative stress induced by H_2_O_2,_ Table S4: Results of HSA–ketoprofen interactions under oxidative stress induced by hydroxyl radical, Table S5: Results of HSA–ketoprofen interactions under oxidative stress induced by Chloramine-T.
