# Supplementary material for: Testing for Ketoprofen Binding to HSA Coated Magnetic Nanoparticles under Normal Conditions and after Oxidative Stress
Source: Molecules. 2020 Apr 22;25(8):1945. doi: 10.3390/molecules25081945 (PMC7221658; doi:10.3390/molecules25081945)
Supplement: Supplementary file 1 [file molecules-25-01945-s001.pdf]

Supplementary materials

Testing for Ketoprofen binding to HSA coated magnetic nanoparticles under normal conditions and after oxidative stress

Marta Ziegler-Borowska[1,*], Kinga Mylkie[1], Pawel Nowak[1], Patryk Rybczynski[1], Adam Sikora[2], Dorota Chelminiak-Dudkiewicz[1] and Anna Kaczmarek-Kedziera[1]

[1] Faculty of Chemistry, Nicolaus Copernicus University in Torun, Gagarina 7, 87-100 Torun, Poland, martaz@umk.pl

[2] Faculty of Pharmacy, Collegium Medicum in Bydgoszcz, Nicolaus Copernicus University in Torun, dr A. Jurasza 2, 85-089 Bydgoszcz, Poland; mmars@cm.umk.pl

* Correspondence: martaz@umk.pl; Tel.: +48-056-611-4916 (M.Z-B.)

1. HPLC analysis

Based on the data containing the declared value of the analyte concentration and the value of the measured signal, a simple calibration was determined, which was then used to determine the marked values. The data obtained in this way were compared with the declared values in order to verify the calibration function.

Table S1. Results of HPLC analysis for ketoprofen calibration curve

Concentration [mg/L]	Peak area	Measured value
1.5625	15713	1.15034
3.125	31926	2.61342
6.25	62480	5.37063
12.5	123109	10.84183
25	272487	24.32180
50	563523	50.58508
100	1132406	101.92146
200	2257901	203.48687
300	3276038	295.36423
400	4467281	402.86279
3.125	32203	2.63841
6.25	63890	5.49786
12.5	127454	11.23392
25	273815	24.44164
50	559139	50.18946
100	1124229	101.18356
200	2237574	201.65255
300	3250830	293.08945
400	4447174	401.04832

3.125	32713	2.68443
12.5	126853	11.17969
25	280259	25.02315
50	577886	51.88120
100	1155206	103.97891
200	2279431	205.42980
300	3267960	294.63526
400	4453636	401.63146

==== Shimadzu LCsolution Analysis Report ====

C:\Documents and Settings\Sterownik1\Pulpit\Adam\CARBON\badania\nowe kulki\Ketoprofen\1A 5.lcd

Acquired by	: Admin
Sample Name	: 1A 5
Sample ID	: 1A 5
Tray#	: 1
Vail #	: 1
Injection Volume	: 5 uL
Data File Name	: 1A 5.lcd
Method File Name	: method.lcm
Batch File Name	: bacz.lcb
Report File Name	: Default.lcr
Data Acquired	: 2018-02-25 12:46:06
Data Processed	: 2018-02-25 12:56:09

<Chromatogram>

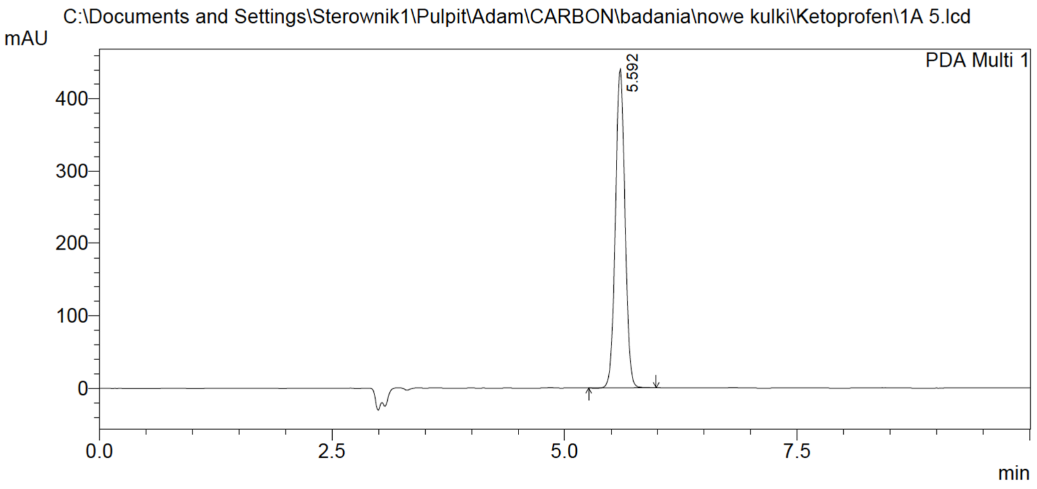

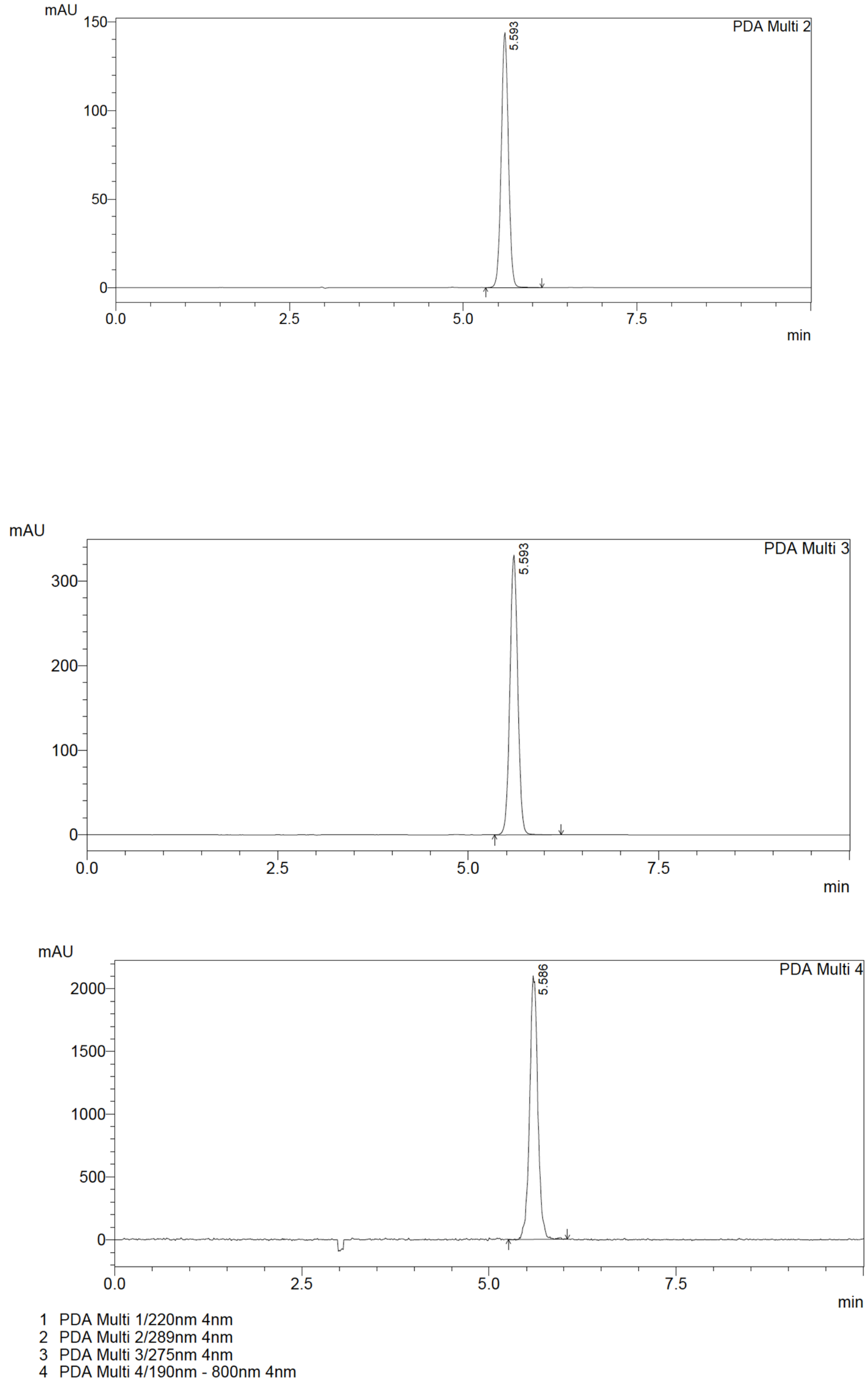

Figure S1. Ketoprofen chromatograms

2. HSA -ketoprofen interaction results

Table S2. Results of ketoprofen interaction with HSA without oxidative stress

Nanoparticles	m_0, [mg]	m_s, [mg]	bounded ketoprofen [%]	bounded ketoprofen [mg]	HSA amount [g]	b [mg/g HSA]
**free HSA**	0.02288	0.01024	55.24	0.01264	0.00166	7.60
**Fe_3O_4 -CS(Glu)-HSA**	0.02288	0.01713	25.12	0.00575	0.00076	7.60
**Fe_3O_4 -CS(SqA)-HSA**	0.02288	0.01686	26.29	0.00602	0.00079	7.60
**Fe_3O_4-CSEt(NH_2)-HSA**	0.02288	0.01148	49.84	0.01140	0.00150	7.60
**Fe_3O_4-CSEt(NH_2)$_3$-HSA**	0.02288	0.00692	69.77	0.01596	0.00210	7.60
**Fe_3O_4-AS-HSA**	0.02288	0.01032	54.91	0.01256	0.00165	7.60

Table S3. Results of HSA -ketoprofen interactions under oxidative stress induced by H_2O_2

Nanoparticles	m_0, [mg]	m_s [mg]	bounded ketoprofen [mg]	b [mg/g HSA]
**free HSA**	0.02288	0.01283	0.01005	6.04
**Fe_3O_4 -CS(Glu)-HSA**	0.02288	0.01826	0.00462	6.12
**Fe_3O_4 -CS(SqA)-HSA**	0.02288	0.01807	0.00481	6.08
**Fe_3O_4-CSEt(NH_2)-HSA**	0.02288	0.01373	0.00915	6.10
**Fe_3O_4-AS-HSA**	0.02288	0.01284	0.01004	6.08
**Fe_3O_4-CSEt(NH_2)$_3$-HSA**	0.02288	0.01005	0.01283	6.11

Table S4. Results of HSA -ketoprofen interactions under oxidative stress induced by hydroxyl radical

Nanoparticles	m_0 [mg]	m_s [mg]	bounded ketoprofen [mg]	b [mg/g HSA]
**free HSA**	0.02288	0.01165	0.01123	6.75
**Fe_3O_4 -CS(Glu)-HSA**	0.02288	0.01761	0.00528	6.98
**Fe_3O_4 -CS(SqA)-HSA**	0.02288	0.01769	0.00520	6.56
**Fe_3O_4-CSEt(NH_2)-HSA**	0.02288	0.01280	0.01008	6.72
**Fe_3O_4-AS-HSA**	0.02288	0.01164	0.01124	6.80
**Fe_3O_4-CSEt(NH_2)$_3$-HSA**	0.02288	0.00864	0.01424	6.78

Table S5. Results of HSA -ketoprofen interactions under oxidative stress induced by Chloramine-T

Nanoparticles	m_0 [mg]	m_s [mg]	bounded ketoprofen [mg]	b [mg/g HSA]
**free HSA**	0.02288	0.01623	0.00665	4.00
**Fe_3O_4 -CS(Glu)-HSA**	0.02288	0.01980	0.00308	4.07
**Fe_3O_4 -CS(SqA)-HSA**	0.02288	0.01975	0.00313	3.95
**Fe_3O_4-CSEt(NH_2)-HSA**	0.02288	0.01683	0.00605	4.03
**Fe_3O_4-AS-HSA**	0.02288	0.01628	0.00660	3.99
**Fe_3O_4-CSEt(NH_2)$_3$-HSA**	0.02288	0.01446	0.00842	4.01
